# Supplementary material for: Structural and Biochemical Characterization of Silver/Copper Binding by Dendrorhynchus zhejiangensis Ferritin
Source: Polymers (Basel). 2023 Mar 3;15(5):1297. doi: 10.3390/polym15051297 (PMC10007213; doi:10.3390/polym15051297)
Supplement: Supplementary file 1 [file polymers-15-01297-s001.zip › polymers-2188222-supplementary.pdf]

Table S1. X-ray diffraction data collection and refinement statistics.

**Crystal parameters**	**Ag^+-DzFer**	**Cu^{2+}-DzFer**
**Data Collection**		
Beam line	SSRF-BL02U1	SSRF-BL18U1
Wavelength (Å)	0.979	0.979
Space group	I432	I432
Cell dimensions		
a, *b*, *c* (Å)	148.21, 148.21, 148.21	151.01, 151.01, 151.01
α, β, γ (°)	90, 90, 90	90, 90, 90
Resolution (Å)†	104.80-1.90 (2.00-1.90)	37.75-2.26 (2.34-2.26)
No. of observed reflections†	803649	453153
No. of unique reflections†	22119	14068
Completeness (%)†	100.0 (100.0)	99.9 (100.0)
Mean I/sigma(I)†	12.5 (2.9)	7.9 (0.6)
Multiplicity†	36.3 (35.3)	32.2 (33.1)
Wilson *B* factor (Å)	30.9	28.9
R_{merge}†	0.260 (1.752)	0.219 (0.595)
$CC_{1/2}$†	0.995 (0.924)	0.997 (0.988)
**Refinement**		
Program	PHENIX/REFMAC5	PHENIX/REFMAC5
R_{work}/R_{free}	0.151/0.190	0.164/0.226
Mean temperature factor (Å^2)	36.0	33.0
No. of non-H atoms	1585	1534
Protein	1375	1366
Water	193	155
Ligands	17	13
Average *B* value (Å^2)		
Protein	33.0	30.7
Water	43.9	39.0
Ligands	66.7	111.3
R.m.s. deviations		
Bond lengths (Å)	0.007	0.007
Bond angles (°)	0.847	0.811
Ramachandran plot, residues in		
Favoured region (%)	98.2	98.2
Allowed region (%)	1.8	1.8
Outlier region (%)	0.0	0.0
Clashscore	4.40	2.97
**PDB code**	8GY1	8HCT

†Values in parentheses correspond to the highest resolution shell.

Table S2. The percentage contents of secondary structure of DzFer at varying pH conditions (pH 2–12).

Sample	α-helix	β-sheet	β-turn	random coil
DzFer (pH 2)	62.37±0.80	22.3±2.4	10.57±1.40	4.73±0.50
DzFer (pH 4)	78.8±0.3	0	21.2±0.3	0
DzFer (pH 6)	82.5	0	17.5	0
DzFer (pH 8)	90.8±0.2	0	9.2±0.2	0
DzFer (pH 10)	88.67±0.10	0	11.33±0.10	0
DzFer (pH 12)	33.83±2.30	53.0±7.3	5.0±4.8	8.13±1.20

Table S3. The composition ratios of secondary structure of DzFer, Ag^{+}-DzFer, and Cu^{2+}-DzFer.

Sample	α-helix	β-sheet	β-turn	random coil
DzFer	90.8±0.2	0	9.2±0.2	0
Ag^{+}-DzFer	73.8±0.8	0	26.1±0.8	0
Cu^{2+}-DzFer	86.6±0.3	0	13.3±0.3	0

Table S4. The percentages of secondary structure of DzFer, Ag^{+}-DzFer, and Cu^{2+}-DzFer after Fe^{2+} ion uptake.

Sample	α-helix	β-sheet	β-turn	random coil
DzFer+Fe^{2+}	84.5±0.7	0	15.5±0.7	0
Ag^{+}-DzFer+Fe^{2+}	72.2±0.7	0	27.7±0.7	0
Cu^{2+}-DzFer+Fe^{2+}	85.7±0.4	0	14.3±0.4	0

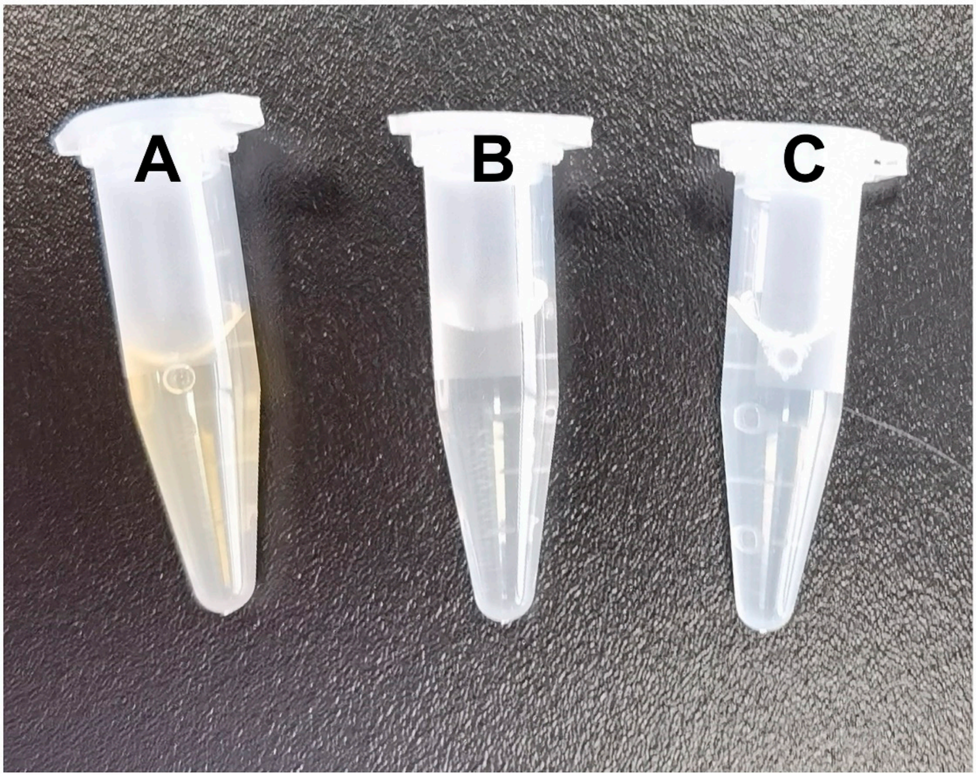

Figure S1. Solution images of DzFer with the concentrations of 5 mg/mL **(A)** and 1 mg/mL **(B)** as well as binding buffer (25 mM Tris–HCl pH 8.0, 150 mM NaCl) **(C)**.

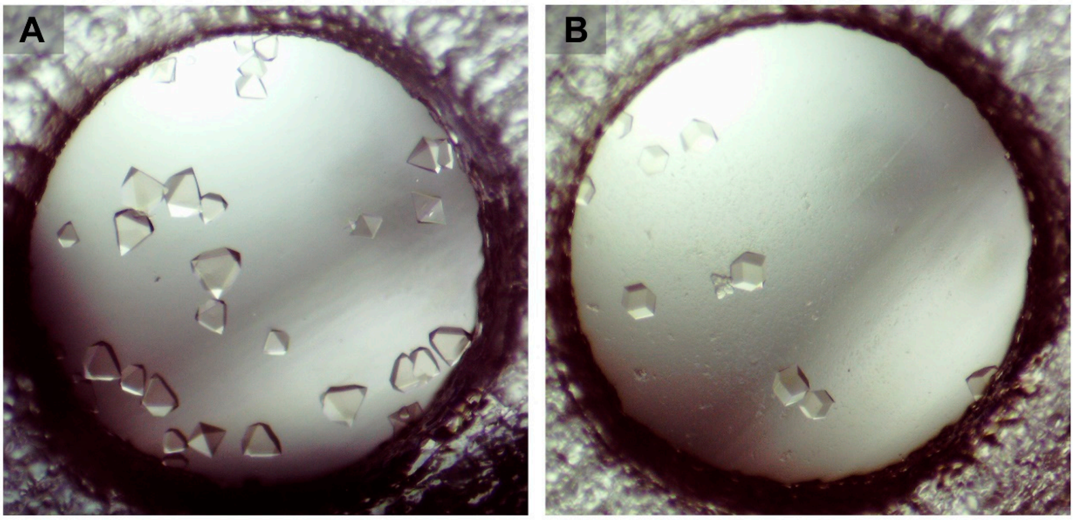

Figure S2. Crystals of DzFer **(A)** and Cu^{2+}-DzFer **(B)**.

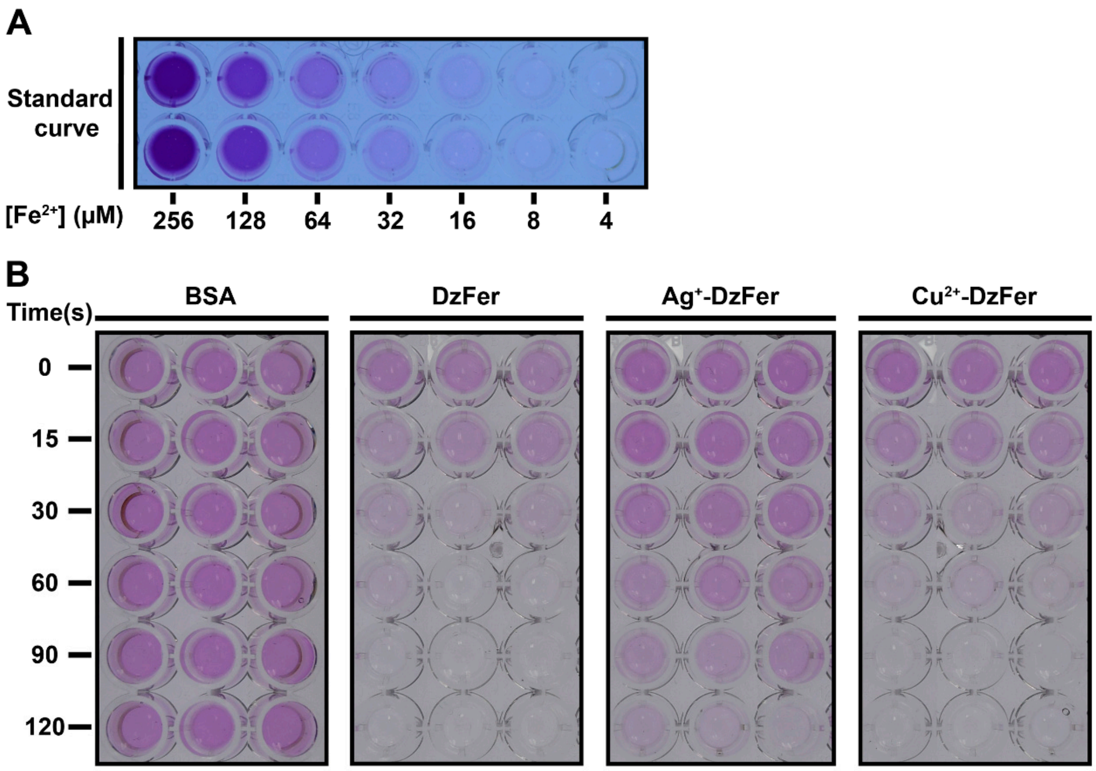

Figure S3. **(A)** A standard curve was drawn based on reading the optical density value at 560 nm by adding ferrozine into known serially diluted different Fe^{2+} concentrations; **(B)** The color reaction by monitoring the ferrozine–Fe^{2+} complexes at different time points revealed the protein samples oxidating Fe^{2+} into Fe^{3+}.

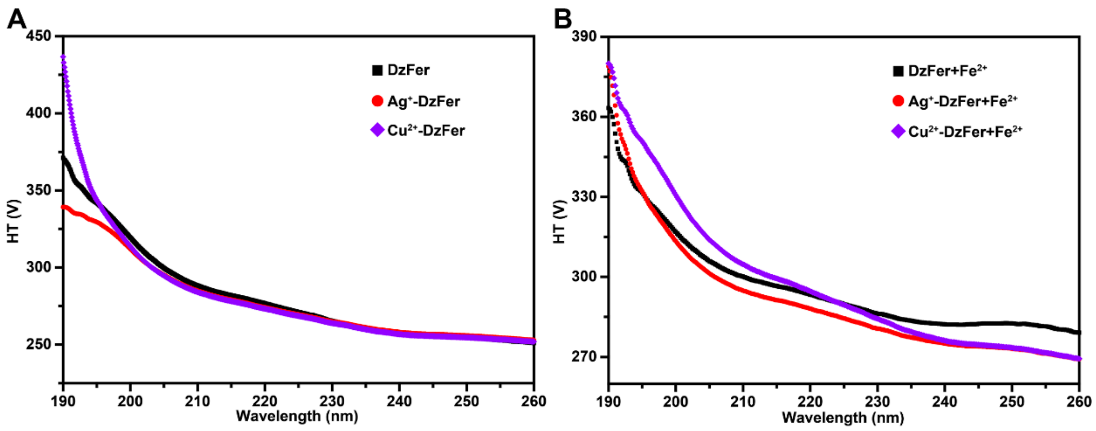

Figure S4. The recorded signals of the high-tension voltage (HT) during the CD measurement. **(A)** The HT traces of DzFer, Ag^{+}-DzFer, and Cu^{2+}-DzFer proteins; **(B)** The HT traces of DzFer+Fe^{2+}, Ag^{+}-DzFer+Fe^{2+}, and Cu^{2+}-DzFer+Fe^{2+} proteins.

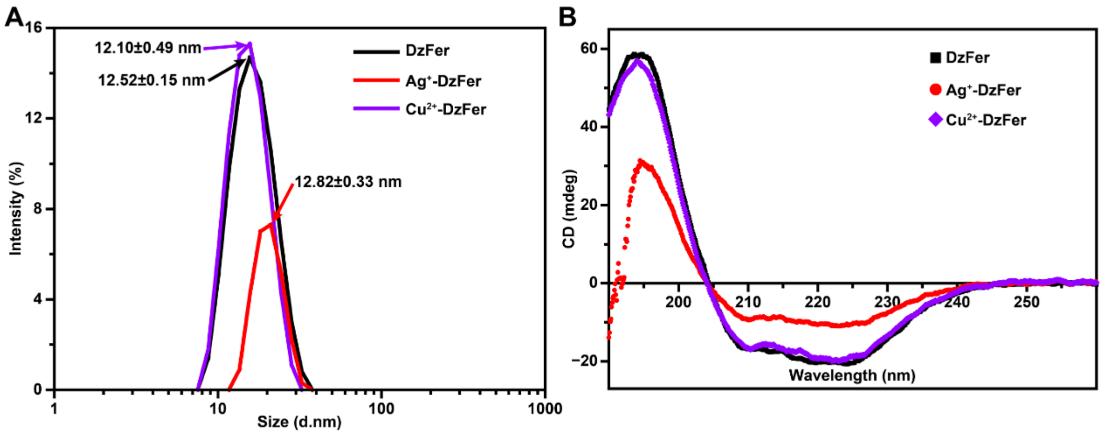

Figure S5. **(A)** DLS analysis of DzFer and metal-bound DzFer; **(B)** CD spectra of DzFer, Ag^{+}-DzFer, and Cu^{2+}-DzFer proteins.

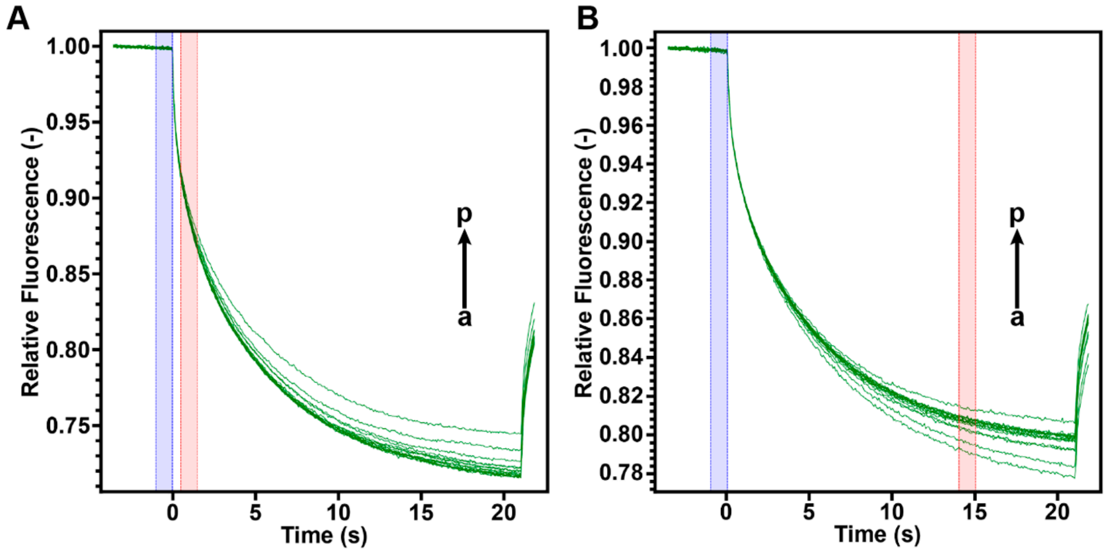

Figure S6. MST-measurements with DzFer and Ag^{+} or Cu^{2+}, respectively. **(A)** MST time traces of fluorescently labeled DzFer (approx. 200 nM) in the presence of different concentrations of Ag^{+} (from 0.275 nM to 9 μM, for curves a to p, respectively); **(B)** MST time traces of fluorescently labeled DzFer (approx. 200 nM) in the presence of different concentrations of Cu^{2+} (from 0.61 nM to 20 μM, for curves a to p, respectively). Scans were recorded at 20% excitation power at 25 °C.

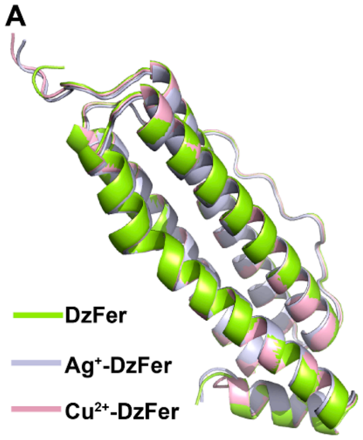

B

	DzFer (168 residues)	Ag^+-DzFer (169 residues)	Cu^{2+}-DzFer (169 residues)
α-helix	75.6	75.1	74.6
β-sheet	0.0	0.0	0.0
β-turn	9.5	7.7	9.4
random coil	14.9	17.2	16.0

Figure S7. (A) The structural superposition of DzFer, Ag^+-DzFer, and Cu^{2+}-DzFer subunits; **(B)** The percentages of the secondary structure assignments based on the known crystal structures of DzFer (PDB ID: 7EMK), Ag^+-DzFer (PDB ID: 8GY1), and Cu^{2+}-DzFer (PDB ID: 8HCT).

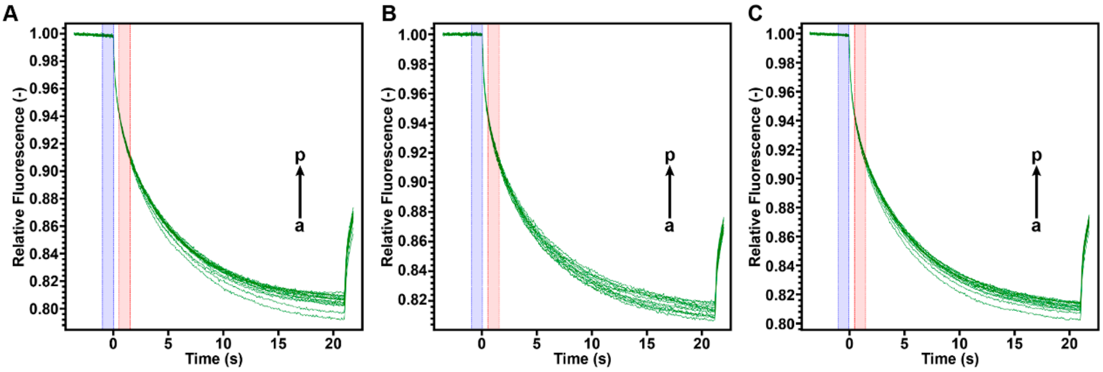

Figure S8. MST time traces of fluorescently labeled protein samples (approx. 200 nM), e.g., DzFer **(A)**, Ag^+-DzFer **(B)**, and Cu^{2+}-DzFer **(C)**, in the presence of different concentrations of Fe^{2+} (from 0.61 nM to 20 μM, for curves a to p, respectively). Scans were recorded at 20% excitation power at 25 °C.
